# 

**Published:** 1870-03

**Authors:** 


*Southern Dental Association.*—This Association will hold its second annual meeting in New Orleans on the 2nd Wednesday of April next. There is every reason to believe that this meeting will be attended by a very large number of the dentists of the Southern States, and prove highly interesting and instructive.

We trust all will attend who can by any possible means make arrangements to do so, as we feel certain they will be well received by the members of the profession in New Orleans, and everything done to add to their comfort and enjoyment, and make their visit to this beautiful city a pleasant one.

